# Effects of the morbid obesity and skin incision choices on surgical outcomes in patients undergoing total abdominal hysterectomy

**DOI:** 10.4274/tjod.67864

**Published:** 2016-12-15

**Authors:** Ebru Ersoy, Özlem Evliyaoğlu, Okyar Erol, Ali Özgür Ersoy, Mehmet Akif Akgül, Ali Haberal

**Affiliations:** 1 Etlik Zübeyde Hanım Women’s and Children’s Health Education and Research Hospital, Clinic of Obstetrics and Gynecology, Ankara, Turkey; 2 Zekai Tahir Burak Women’s Healthcare Training and Research Hospital, Clinic of Obstetrics and Gynecology, Ankara, Turkey

**Keywords:** morbid obesity, gynecologic surgery, complications, wound infection, hysterectomy

## Abstract

**Objective:**

This study aimed to evaluate the effect of obesity on surgical outcomes in patients who underwent gynecologic surgery.

**Materials and Methods:**

In total, we evaluated 132 patients who underwent total abdominal hysterectomy with or without salpingo-oophorectomy for benign gynecologic procedures at our tertiary referral gynaecology clinic.

**Results:**

The non-morbid obese group [body mass index (BMI) <40 kg/m^2^] included 94 patients, and the morbid obese group (BMI ≥40 kg/m^2^) included 38 patients. The perioperative outcomes of the groups were compared. The mean operative time was significantly longer for morbid obese patients than non-morbid obese patients (p<0.05). Estimated blood loss, the need for blood transfusion, postoperative hemoglobin values, and the need for an intraabdominal drain were similar between the groups. Early and late postoperative complications were significantly more frequent in the morbid obese group than the other group (p<0.05, for each). Early postoperative complications in patients who underwent vertical skin incision were significantly more frequent than in patients who underwent pfannenstiel incision (p<0.05). Late complications were comparable between the two types of skin incision.

**Conclusion:**

Morbid obesity significantly increases the mean operative times and the postoperative complication rates of abdominal hysterectomy operations.

## INTRODUCTION

Obesity is a common health problem and is defined as a body mass index (BMI) of 30 kg/m^2^ or greater; morbid obesity is defined as a BMI ≥40 kg/m^2(1)^. In the United States of America, the incidence of obesity among adults has been reported to increase two-fold during the past decade^(2)^. In European and Mediterranean regions, the incidence of overweight (i.e., BMI between 25 and 30 kg/m^2^) and obesity have increased during recent decades, regardless of the level of development^(3,4,5)^.

Obesity is associated with an increased risk of diabetes mellitus, polycystic ovary syndrome, hypertension, dyslipidaemia and coronary heart diseases^(6)^. Obese patients have a significantly higher risk of postoperative myocardial infarction, surgical site infections, nerve injury, and urinary infection. Obesity is an independent risk factor for perioperative morbidity, and morbid obesity is a risk factor for perioperative mortality^(7,8)^.

Laparotomy is frequently used in surgical procedures for gynecologic disorders, such as myomas, adnexal masses, and tubo-ovarian abscess^(9)^. Due to the increasing weight of the population, we encounter more obese patients in our gynecologic practice, even if we try to keep them away from surgical interventions. Physicians should avoid surgery (especially open abdominal surgery) in obese and morbid obese patients as well as they can. In laparotomies for gynecologic diseases, we generally use two types of skin incision. Pfannenstiel incisions are preferred because this type of incision provides adequate vision in the pelvic area and has good cosmetic results. However, a vertical skin incision is sometimes preferred for a giant myoma or a giant adnexal mass^(10)^.

There are insufficient data in the literature regarding complication rates arising from morbid obesity and the chosen type of skin incision in benign gynecologic hysterectomies. Thus, we aimed to evaluate the effect of morbid obesity and the type of incision on complication rates among patients who underwent surgery in our clinic.

## MATERIALS AND METHODS

This retrospective study was performed between June 2006 and February 2007 at Etlik Zübeyde Hanım Women’s Health Training and Research Hospital, which is a tertiary referral center in Ankara. Ethical approval for our study was obtained from the Local Ethics Committee. The research was completed in accordance with the Helsinki Declaration^(11)^. It included adult women who attended to our hospital for a benign gynecologic pathology and underwent total abdominal hysterectomy. Patients were excluded if they had any malignant disease, skin disease (such as psoriasis), autoimmune disease or were immunosuppressed. All patients were Caucasian Turkish women with no history of alcohol or drug use before and after surgery. We collected data from hospital records and patient files, and the same author called all patients to inquire about the presence of any complications within 30 days of surgery or more lately after discharge from the hospital. The subject characteristics and demographics were analyzed. Demographic preexisting variables included age, BMI, obstetric history, tobacco use, history of any previous abdominal surgery, and presence of any systemic comorbidity. BMI (kilograms per square meter) was calculated using the patient’s height and weight. The subjects were divided into two BMI categories according to the World Health Organization classification system^(12)^. The morbid obesity group included consecutive subjects who had a BMI ≥40 kg/m^2^, and the non-morbid obesity group included consecutive subjects who had a BMI lower than 40 kg/m^2^.

Moreover, we classified the same samples into sub-groups in terms of skin incision types: vertical incision or pfannenstiel incision. All surgeries were performed by the same surgical team using a standardized technique. We performed surgery for benign gynecologic disorders such as myoma uteri, adnexal mass, persistent uterine hemorrhage, and tubo-ovarian abscess, and performed total abdominal hysterectomies with or without salpingo-oophorectomy for all subjects, based on the indication for surgery. Operative time (minutes), preoperative and postoperative haemoglobin (Hb) values

(g/dL), estimated blood loss (EBL) (mL), requirement for blood product transfusion, length of hospital stay (days), and drain presence were recorded for each patient. A surgical drain was placed into the abdominal space based on the preference of the surgeon. When the thickness of the subcutaneous layer was greater than 2 centimetres, the subcutaneous space was closed with 2/0 poliglactic acid-poliglactin (vicryl). Subcutaneous drains were not used in any case. Cefazolin (1 gram) was used as the primary preoperative prophylaxis; clindamycin was used for patients with a history of penicillin allergy. Both drugs were administered intravenously, and additional doses were administered when the operation lasted longer than two hours. Prophylaxis for venous thromboembolic events was performed according to the chest guidelines released by the American College of Chest Physicians^(13)^. Early postoperative complications were defined as complications that occurred during the operation or within 30 days of the surgery. Late postoperative complications were defined as complications that occurred more than 30 days after the surgery. The operative time was defined as the time from the first skin incision to the final closure of the skin incision. Wound complications were defined as a subcutaneous infection or hemorrhage requiring surgical debridement and repair. Superficial skin separation was not considered as a wound complication. Wound infection was defined as a wound with purulent or serous drainage in combination with tissue warmth, erythema, and increasing tenderness. The primary outcome measure was defined as the presence of any early and/or late complications in morbid obese patients. The presence of complications with any incision type was also recorded. Continuous variables were recorded as the mean ± standard deviation or the median and interquartile ranges, and categorical variables were reported as frequencies and column percentages. The normality of the variables was analyzed using the Kolmogorov-Smirnov test. Student’s t-test was used to compare normally distributed continuous variables, and the Mann-Whitney U test was used to compare non-normally distributed continuous variables. The chi-square test or Fisher’s exact test (when chi-square test assumptions do not hold due to low expected cell counts), where appropriate, was used to compare categorical variables. Two-sided p-values were considered statistically significant at p<0.05. Statistical analyses were performed using the statistical package SPSS version 17.0 for Windows (SPSS Inc., Chicago, IL, USA).

## RESULTS

### Patient characteristics

The demographic characteristics of a total of 132 patients are listed in Table 1. Patients in the study group with morbid obesity were significantly older and had a significantly higher prevalence of comorbidities than patients in the non-morbid obesity group. The two groups were similar with respect to tobacco use, previous abdominal surgery, and menopause status.

### Perioperative outcomes

The surgical data of the two groups are shown in Table 2. Skin incision types were comparable between the two groups. Vertical incisions were midline incisions below the umbilicus in all cases except one, in which the incision also extended above the umbilicus. The pre-operative Hb values were significantly lower, and the mean operative time (minutes) was significantly longer in the morbid obesity group than the non-morbid obesity group. EBL, the requirement for blood transfusion, post-operative Hb values, and the presence of an abdominal drain were similar between groups. The mean length of hospital stay was longer in the group with morbid obesity than the other group (Table 2).

### Complications

In our study, no intraoperative complications were observed, but there were some post-operative complications. We assessed the effect of morbid obesity on post-operative complications (Table 3). Early and late complications were significantly more frequent in the morbid obesity group than the non-morbid obesity group. We observed no wound cellulitis or fascial dehiscence in any patients.

We also compared the complications between incision types (i.e., vertical and pfannenstiel). Patients with a vertical incision had significantly more early complications (n=9; 23.1%) than patients with a pfannenstiel incision (n=3; 3.2%) (p<0.05). Late complications occurred in 4 subjects (10.3%) in the vertical incision group and 3 subjects (3.2%) in the pfannenstiel incision group. No significant differences in late complications were observed between the two types of skin incision (Table 4).

## DISCUSSION

We assessed the consequences of morbid obesity in our gynecologic surgery practice. Morbidly obese patients had more comorbidities, longer operation times, longer hospital stays, more early and late complications than the other group of patients. History of previous abdominal surgery, tobacco use, menopause status, and skin incision types were comparable between the two groups, so these variables are not likely to affect differential complication rates. Hysterectomy is the most common major gynecologic surgery^(14)^. To our knowledge, no recent study in the literature has evaluated complications related to obesity and chosen skin incision types in benign gynecologic practice. Some studies investigated obesity and gynecologic cancer surgery^(15,16)^. However, benign gynecologic operations are performed more frequently than gynecologic cancer operations. The pelvic area where we performed surgery was deeper in morbidly obese patients, and the surgeons had difficulty obtaining an adequate field of vision; thus, a longer operative time was needed in morbidly obese patients. A study by Kodama et al.^(16)^ demonstrated that a longer operative time was an independent predictor of the incidence of early postoperative complications. The results of our study confirmed this finding.

A previous study stated that obese women had greater blood loss, longer operative times and hospital stays, and an increased rate of wound infection in comparison to non-obese patients^(17)^. However, we obtained different results concerning blood loss, because we observed no differences in EBL, postoperative Hb or the need for blood transfusion between the two groups. In our daily practice, we attempt to mobilize morbidly obese patients as early as possible, direct them to use antiembolic stockings to prevent thromboembolic events, and encourage them to return to their daily lives. The utmost importance of thromboembolic prophylaxis in morbid obese patients was denoted well in a systematic review by Hodges et al.^(18)^. Increased venous stasis, which has already been provoked by morbid obesity and major surgery, has been cited as responsible for venous thromboembolism^(13)^. In our study, no intraoperative complications were detected. We can attribute it to our experienced surgical team. A recent study performed by the Gynecologic Oncology Group LAP2 reported no differences in intra-operative complications between obese and non-obese patients^(19)^. Another study found that obese patients did not experience an increased risk of serious morbidity after vaginal and abdominal hysterectomy compared with normal weight women^(20)^. These studies compared obese and non-obese patients; however, we studied surgical complications in morbidly obese and non-morbidly obese patients. When we evaluated the groups with respect to postoperative complications, significantly higher rates of early and late complications were observed in the morbidly obese group. This finding was not surprising. In most studies, morbidly obese patients have been found to have a greater risk of postoperative complications than non-obese patients^(21)^. A study about vaginal procedures in overweight patients found no difference in postoperative complications between the study and the control groups^(22)^. However, the sample specifications and surgical procedures in that study were different from ours. In a study by Geppert et al.^(23)^ robotic-assisted laparoscopy and open surgery for benign hysterectomy indications in obese and morbidly obese patients were compared in terms of surgical outcomes. As a result, the complication rate in the robotic surgery group was found lower than the open surgery group. However, no control groups to date have comprised normal BMI or overweight (25 kg/m^2^< BMI <30 kg/m^2^) patients. Therefore, that study could not have indicated the disadvantages of obesity on surgical outcomes^(23)^. Clinical surveys of other surgical fields, such as orthopedics and gastroenterologic surgery concluded that surgery was safe for obese patients, except in emergency operations^(24,25)^. Our study demonstrated that one-third of 38 patients with morbid obesity (13 patients) had one early or late complication. Most of the early complications were wound infections. In a recent study, increasing BMI was found associated with increased operative time and surgical site infections in patients undergoing abdominal hysterectomy. The results of that study are consistent with ours, but our study has different characteristics of indications for hysterectomy and salpingo-oophorectomy status^(26)^. We wondered whether fewer wound infections would occur if we used a subcutaneous drain. A previous study reported that subcutaneous drains and prophylactic antibiotics were recommended to minimise wound disruption^(27)^. However, when we reviewed the literature, we found that for patients with more than 3 cm of subcutaneous fat, the use of a subcutaneous drain was not effective for the prevention of superficial wound disruption^(9)^. We do not believe that surgical drains would lead to fewer wound infections because the development of a surgical site infection after a surgical procedure depends on the interaction between the host, the microorganism, and the surgical environment. In our study, no significant differences were observed between the non-morbidly obese and morbidly obese groups in terms of skin incision type. We investigated the complication rates according to the type of skin incision. The frequency of late complications was comparable between the two types of skin incision, but the early complication rate was significantly higher in the vertical incision group. A previous study of cesarean section revealed a higher incidence of wound complications in morbidly obese patients, and indicated that a vertical skin incision was associated with a higher rate of wound complications than a transverse incision^(28)^. Likewise, two other studies showed that in comparison to low transverse incisions, vertical skin incisions were associated with increases in postoperative pain, postoperative atelectasis, superficial wounds, and fascial dehiscence^(10,29)^.

No patients in our study had major complications such as massive bleeding or bowel injuries; this finding may be have been caused by the limited number of patients in the study. Further studies with a larger sample sizes may generate different findings. In our study, gynecologic operations were performed using an abdominal incision only; we did not include any vaginal or laparoscopic gynecologic surgeries. There are some limitations to our study. Absence of sample size analysis is one of them. Owing to the cross-sectional study design, all of the consecutive patients who attended our hospital and fulfilled the inclusion criteria between the mentioned dates were recruited for the study. The strengths of our study include the investigation of the effects of morbid obesity and skin incision types on surgical outcomes in major gynecologic surgery.

## CONCLUSION

Hysterectomy with or without salpingo-oophorectomy is significantly associated with early and late postoperative complications in morbidly obese patients. Early complications in patients undergoing abdominal hysterectomy with a vertical incision were encountered more frequently than in patients undergoing hysterectomy with a transverse incision. Wound infection was seen as an early complication in predominantly morbidly obese patients. Further studies that compare larger samples with more clinical risks and complications may generate different results. Obesity remains an important factor in everyday gynecologic practice.

## Figures and Tables

**Table 1:**
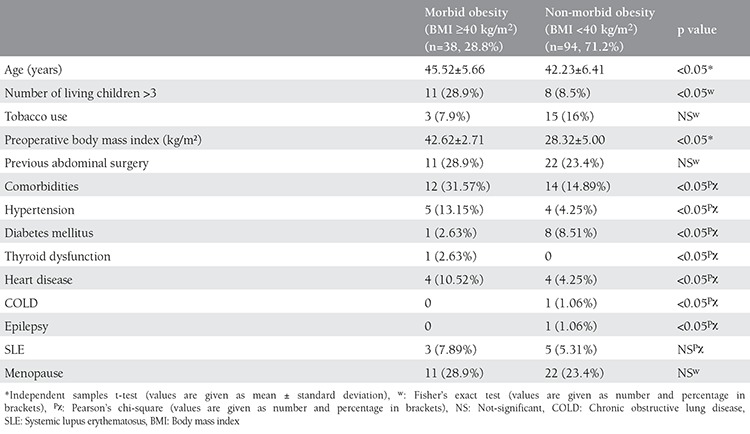
Individual characteristics of the patients in the two groups

**Table 2:**
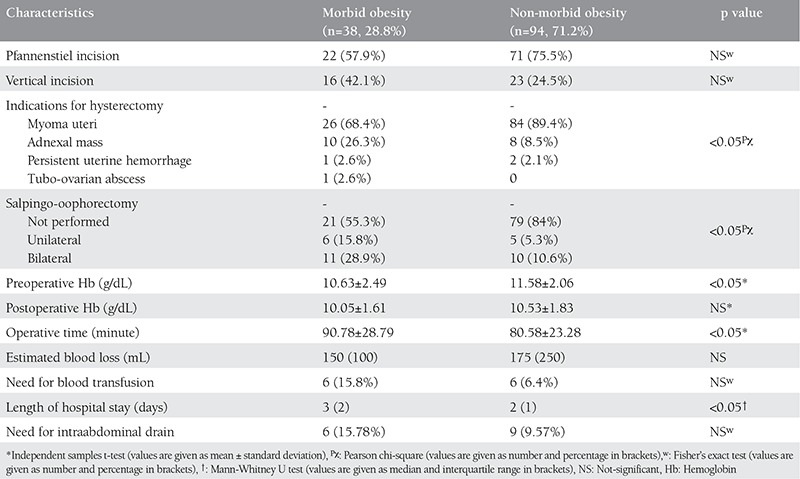
Perioperative characteristics of the two groups

**Table 3:**
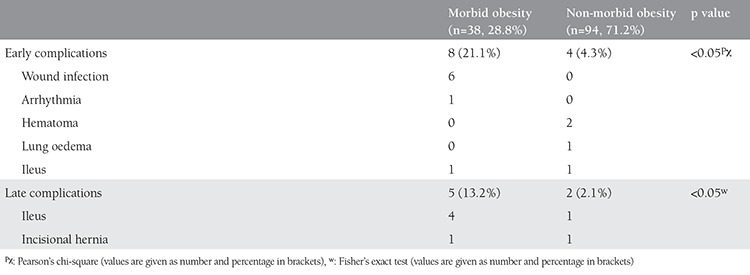
Comparison of post-operative complications between groups

**Table 4:**
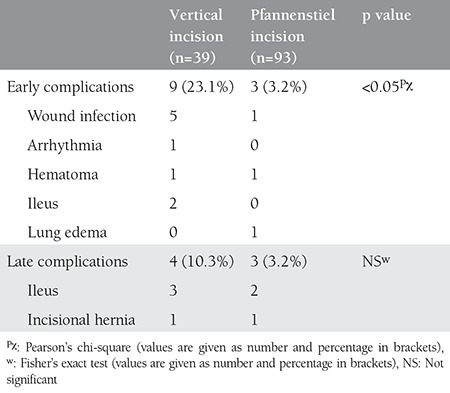
Comparison of early and late complications according to incision type
